# Climate Change Affects Winter Chill for Temperate Fruit and Nut Trees

**DOI:** 10.1371/journal.pone.0020155

**Published:** 2011-05-24

**Authors:** Eike Luedeling, Evan H. Girvetz, Mikhail A. Semenov, Patrick H. Brown

**Affiliations:** 1 World Agroforestry Centre (ICRAF), Nairobi, Kenya; 2 The Nature Conservancy, Seattle, Washington, United States of America; 3 Rothamsted Research, Harpenden, United Kingdom; 4 Department of Plant Sciences, University of California Davis, Davis, California, United States of America; Institut Mediterrani d'Estudis Avançats (CSIC/UIB), Spain

## Abstract

**Background:**

Temperate fruit and nut trees require adequate winter chill to produce economically viable yields. Global warming has the potential to reduce available winter chill and greatly impact crop yields.

**Methodology/Principal Findings:**

We estimated winter chill for two past (1975 and 2000) and 18 future scenarios (mid and end 21st century; 3 Global Climate Models [GCMs]; 3 greenhouse gas emissions [GHG] scenarios). For 4,293 weather stations around the world and GCM projections, Safe Winter Chill (SWC), the amount of winter chill that is exceeded in 90% of all years, was estimated for all scenarios using the “Dynamic Model” and interpolated globally. We found that SWC ranged between 0 and about 170 Chill Portions (CP) for all climate scenarios, but that the global distribution varied across scenarios. Warm regions are likely to experience severe reductions in available winter chill, potentially threatening production there. In contrast, SWC in most temperate growing regions is likely to remain relatively unchanged, and cold regions may even see an increase in SWC. Climate change impacts on SWC differed quantitatively among GCMs and GHG scenarios, with the highest GHG leading to losses up to 40 CP in warm regions, compared to 20 CP for the lowest GHG.

**Conclusions/Significance:**

The extent of projected changes in winter chill in many major growing regions of fruits and nuts indicates that growers of these commodities will likely experience problems in the future. Mitigation of climate change through reductions in greenhouse gas emissions can help reduce the impacts, however, adaption to changes will have to occur. To better prepare for likely impacts of climate change, efforts should be undertaken to breed tree cultivars for lower chilling requirements, to develop tools to cope with insufficient winter chill, and to better understand the temperature responses of tree crops.

## Introduction

Commercially successful cultivation of many fruit and nut trees requires the fulfillment of a winter chilling requirement, which is specific for every tree cultivar [1], [2], [3], [4]. In order to avoid frost damage of sensitive tissue in the cold winters of their regions of origin, trees from temperate or cold climates evolved a period of dormancy during the cold season. After a certain duration of cold conditions (chilling), endodormancy is broken and the tree is ready to resume growth in spring. Chilling requirements vary substantially between species and cultivars from different parts of the world and commercial production of temperate tree crops requires selecting appropriate cultivars for the climatic conditions of the planned production site.

Climate change is likely to affect future winter chill and could have a major impact on the US$ 93 billion global fruit and nut industry (only species with chilling requirements, production statistics for 2005 from ref. [5], currencies converted into 2005 US$ according to ref. [6]). Temperatures are expected to increase in most parts of the world, with minimum temperatures rising most rapidly. This development may compromise the ability of many growers of temperate fruits and nuts to successfully produce the same array of crops as in the past. Climate change effects on winter chill have recently been analyzed for California [7], [8], Germany [9] and high-mountain oases in Oman [10]. While conditions in Germany were relatively stable during the 20^th^ century, winter chill was found to have declined in California and Oman, and this process was expected to continue in the future. The differences between these studies indicate that different growing regions may be differentially impacted, but to date, no estimates are available at a global scale to indicate which regions will maintain adequate winter chill for temperate fruits and nuts in the future. This study aims to fill this knowledge gap and to provide important information needed to evaluate the future viability of fruit and nut growing regions around the world.

Several models have been developed for quantifying winter chill, e.g. the Chilling Hours Model [11], the Utah Model [12] and the Dynamic Model [13], [14]. These models differ greatly in their sensitivity to climate change [15], making the choice of the model a crucial determinant of the predicted extent of climate change effects on winter chill. When using different models for similar climate change scenarios, the Chilling Hours Model and the Utah Model tend to show much stronger decreases in winter chill than the Dynamic Model, especially in warm growing regions [15]. Using the former two models probably overestimates winter chill losses, because several studies have shown the Dynamic Model to be more accurate, especially in subtropical climates [16], [17], [18]. One more study found it to be equal to the Utah Model in Spain [19], and another one reported failure of all models on the Tropical island of Réunion [20]. Calculating ratios between winter chill estimates with different models at warm locations shows large differences, with strong variation and strong temperature dependence [21]. For colder regions, however, such ratios tend to be fairly similar and much less variable [21]. Consequently, the Dynamic Model can be used as a proxy of winter chill in both warm and cold growing regions and, among the common winter chill models, is the one most suitable for a global analysis. The Chilling Hours and Utah Models may produce reasonably accurate results in cold regions, but are not applicable for warmer parts of the world, where their use would produce misleading overestimates of likely impacts of climate change [8]. In this study, we therefore only use the Dynamic Model (for equations, see [21]), which quantifies winter chill in Chill Portions (CP).

We quantified winter chill for the entire terrestrial globe using climate scenarios based on observed daily weather from 4293 weather stations around the world and climate projections from three Global Climate Models (GCMs). Based on this analysis we calculated the safe winter chill (SWC) metric [8], which quantifies the amount of winter chill that is exceeded in 90% of years. This metric is meaningful to fruit and nut producers, because failure to meet chilling requirements in more than 10% of years is likely to render production uneconomical. This analysis identifies important fruit and nut producing areas in the world where SWC has already decreased and is projected to decrease further, and provides a comprehensive assessment of the magnitude of changes to winter chill that will likely occur.

Daily weather records for all weather stations were obtained from the National Climatic Data Center of the United States [22], subjected to a data quality filter, and used to calibrate a weather generator [23], [24]. Daily weather records were then generated for 20 climate scenarios. Two scenarios represented typical climatic conditions in 1975 and 2000. Eighteen future scenarios were generated by extracting future projections from datasets assembled in the ClimateWizard tool [25]. These included statistically downscaled projections with three GCMs (MIROC3.2 (medres), UKMO-HadCM3 and CSIRO-Mk3.0; referred to as MIROC, HADCM3 and CSIRO in the following) and for three IPCC greenhouse gas emissions scenarios (B1 - global curbing of emissions over the 21^st^ century; A1B - emissions leveling off at mid 21^st^ century; and A2 - continually increasing rate of greenhouse gas emissions). Projections were made for the middle (2040–2059) and end (2080–2099) of the 21^st^ century. Idealized daily temperature curves were used for converting daily to hourly weather records and allow calculation of winter chill. For each scenario, 101 years of weather records were generated and the 10% quantile of the resulting distribution of annual winter chill interpreted as Safe Winter Chill. For each scenario, SWC from all stations was spatially interpolated, and winter chill for all scenarios was extracted from the resulting layers for 24 important growing regions around the world.

## Results

**Figure 1:**
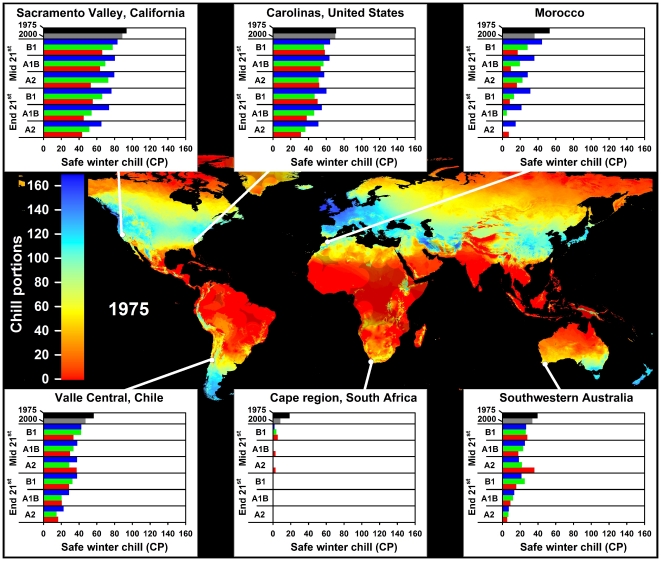
Modeled Safe Winter Chill around the year 1975 (large map), as well as site-specific estimates of Safe Winter Chill for six growing regions and for 20 climate scenarios, representing four points in time (1975, 2000, mid and end 21^st^ century). Future projections include three greenhouse gas emissions scenarios (B1, A1B and A2) and three Global Climate Models (CSIRO - green bars; HADCM3 - blue bars; and MIROC - red bars). Areas that are more than 5° away from the closest weather station are shaded, because interpolated results are unreliable.

In all climate scenarios, estimates of Safe Winter Chill ranged from 0 CP in tropical and very cold regions to about 170 CP in maritime temperate climates of Northwestern Europe (Figs. 1–4). While the overall range of the winter chill distribution did not change much across all scenarios, our results show changes in the global distribution of winter chill, as well as site-specific trends. Because the Dynamic Model does not consider freezing temperatures to be effective for chilling, reduced incidence of frost tends to increase the number of Chill Portions in cold regions. This process is reflected in increasing Safe Winter Chill in cold regions (Fig. 5), which may affect fruit growing regions in Canada, Southern Scandinavia and Eastern Europe (Fig. 4). Decreases are projected for warmer regions, in particular around the Mediterranean Sea (Fig. 6) and in Southwestern North America (Fig. 7), where losses up to 40 CP are expected by the end of the 21st century (Fig. 5). Many warm growing regions are projected to lose most of their winter chill, with South Africa, Southern Australia and Northern Africa particularly affected (Figs. 6 and 8).

**Figure 2 pone-0020155-g002:**
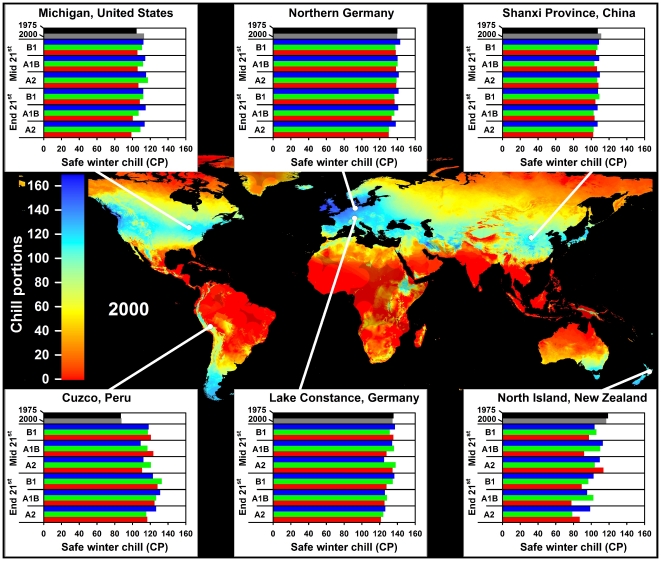
Modeled Safe Winter Chill around the year 2000 (large map), as well as site-specific estimates of Safe Winter Chill for six growing regions and for 20 climate scenarios, representing four points in time (1975, 2000, mid and end 21^st^ century). Future projections include three greenhouse gas emissions scenarios (B1, A1B and A2) and three Global Climate Models (CSIRO - green bars; HADCM3 - blue bars; and MIROC - red bars). Areas that are more than 5° away from the closest weather station are shaded, because interpolated results are unreliable.

**Figure 3 pone-0020155-g003:**
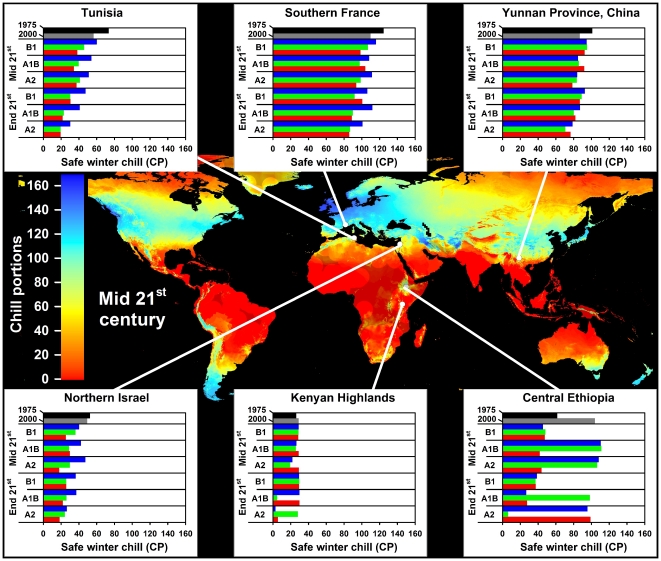
Modeled Safe Winter Chill around the middle of the 21^st^ century averaged over three greenhouse gas emissions scenarios and three Global Climate Models (large map), as well as site-specific estimates of Safe Winter Chill for six growing regions and for 20 climate scenarios, representing four points in time (1975, 2000, mid and end 21^st^ century). Future projections include three greenhouse gas emissions scenarios (B1, A1B and A2) and three Global Climate Models (CSIRO - green bars; HADCM3 - blue bars; and MIROC - red bars). Areas that are more than 5° away from the closest weather station are shaded, because interpolated results are unreliable.

**Figure 4:**
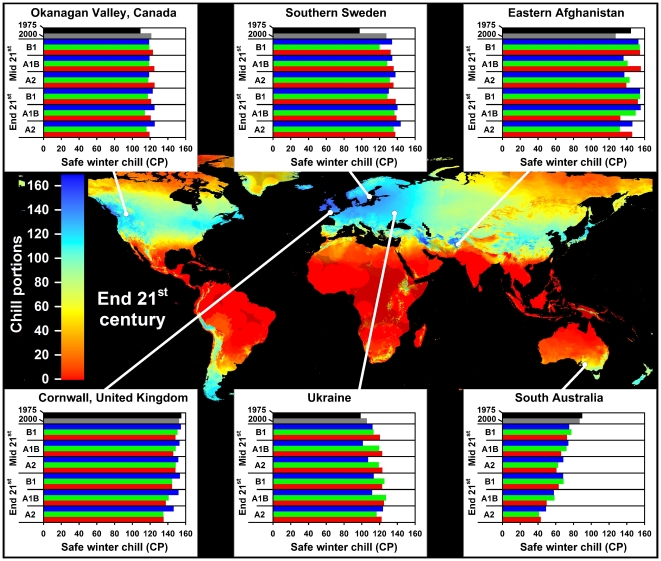
Modeled Safe Winter Chill around the end of the 21^st^ century averaged over three greenhouse gas emissions scenarios and three Global Climate Models (large map), as well as site-specific estimates of Safe Winter Chill for six growing regions and for 20 climate scenarios, representing four points in time (1975, 2000, mid and end 21^st^ century). Future projections include three greenhouse gas emissions scenarios (B1, A1B and A2) and three Global Climate Models (CSIRO - green bars; HADCM3 - blue bars; and MIROC - red bars). Areas that are more than 5° away from the closest weather station are shaded, because interpolated results are unreliable.

**Figure 5:**
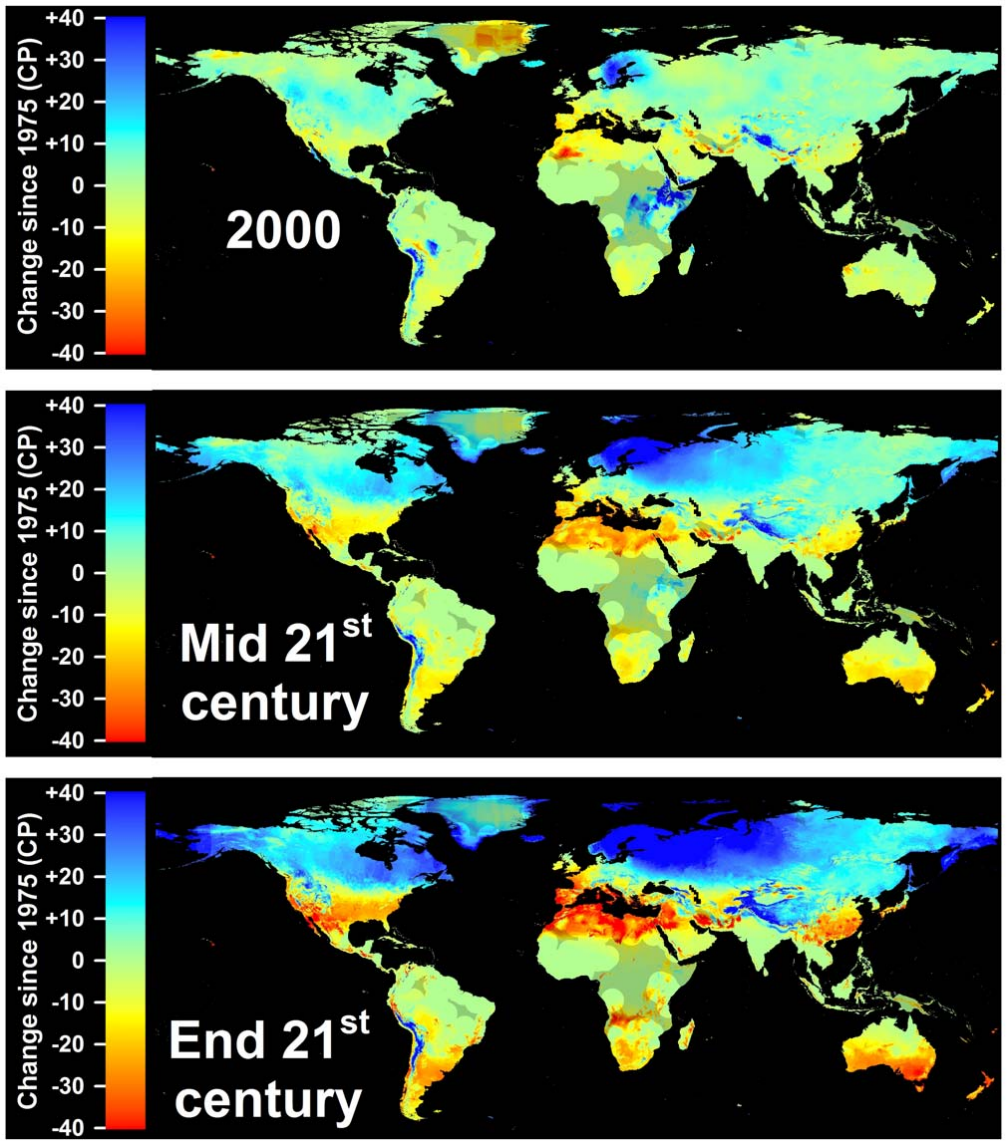
Modeled and projected losses in Safe Winter Chill compared to 1975 for the year 2000 (top), the middle of the 21^st^ century (middle), and the end of the 21^st^ century (bottom). For each point in time, results are averaged over three greenhouse gas emissions scenarios and three Global Climate Models. Areas that are more than 5° away from the closest weather station are shaded, because interpolated results are unreliable.

**Figure 6:**
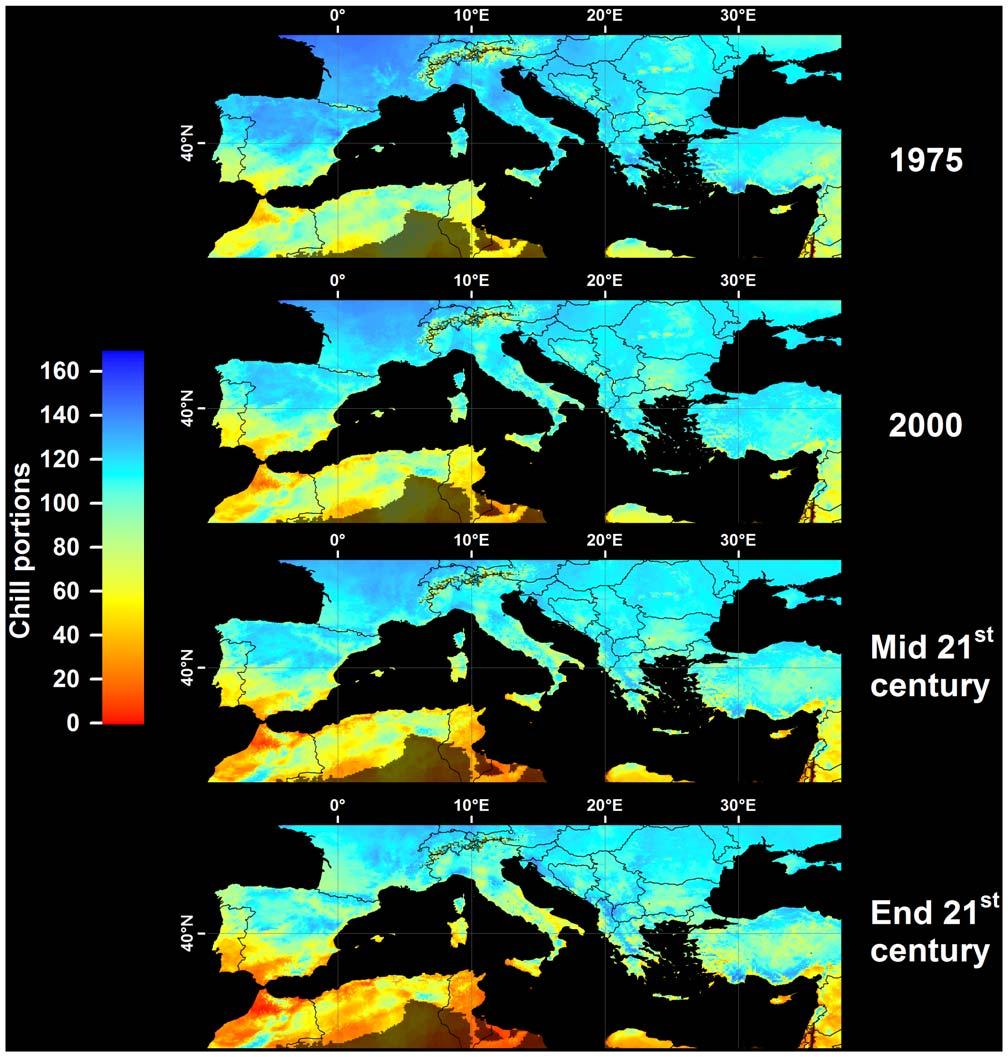
Modeled and projected Safe Winter Chill in the Mediterranean region, for 1975, 2000, the middle of the 21^st^ century (middle), and the end of the 21^st^ century (bottom). For each point in time, results are averaged over three greenhouse gas emissions scenarios and three Global Climate Models. Areas that are more than 5° away from the closest weather station, and areas with mean annual temperatures >20 or <°0C are shaded.

**Figure 7:**
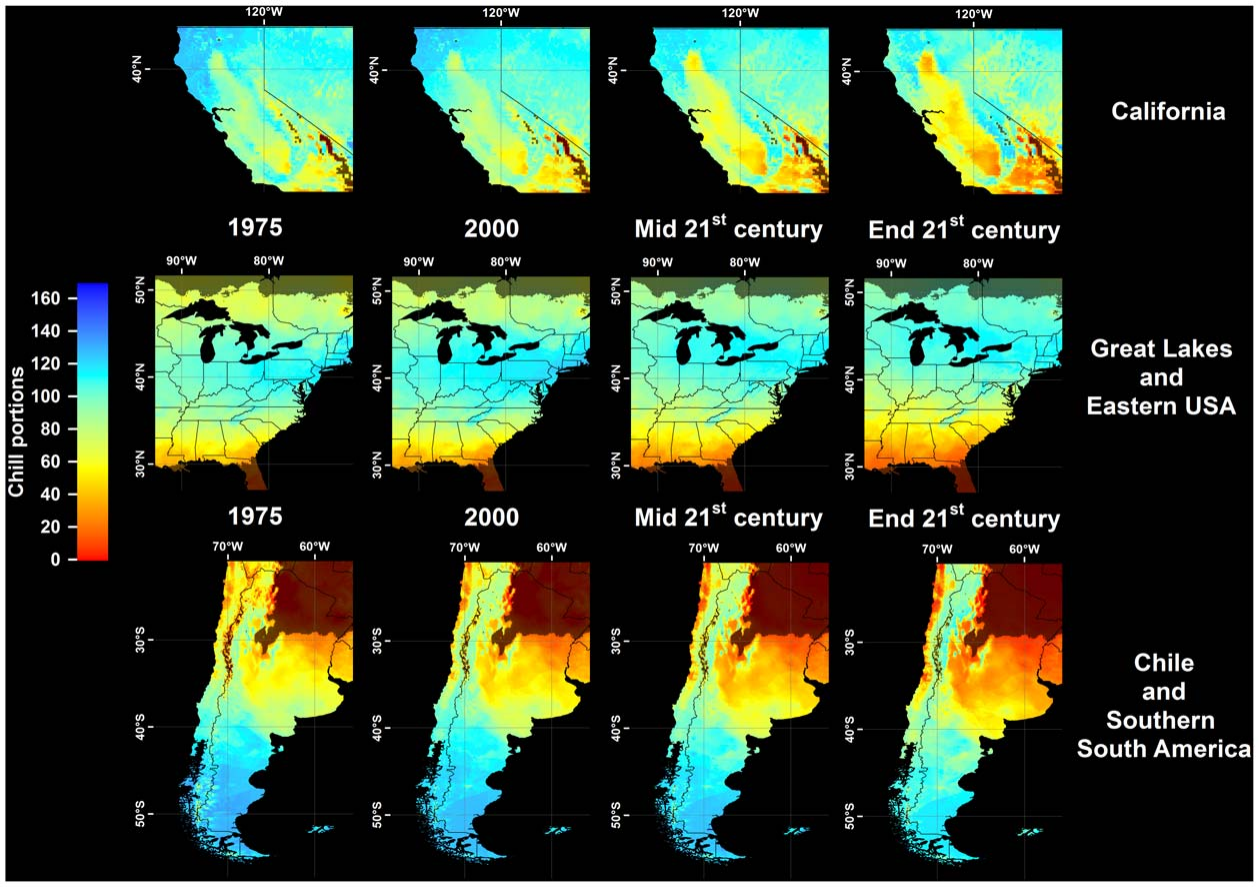
Modeled and projected Safe Winter Chill in California, the Eastern United States and Southern South America, for 1975, 2000, the middle of the 21^st^ century (middle), and the end of the 21^st^ century (bottom). For each point in time, results are averaged over three greenhouse gas emissions scenarios and three Global Climate Models. Areas that are more than 5° away from the closest weather station, and areas with mean annual temperatures >20 or <°0C are shaded.

**Figure 8:**
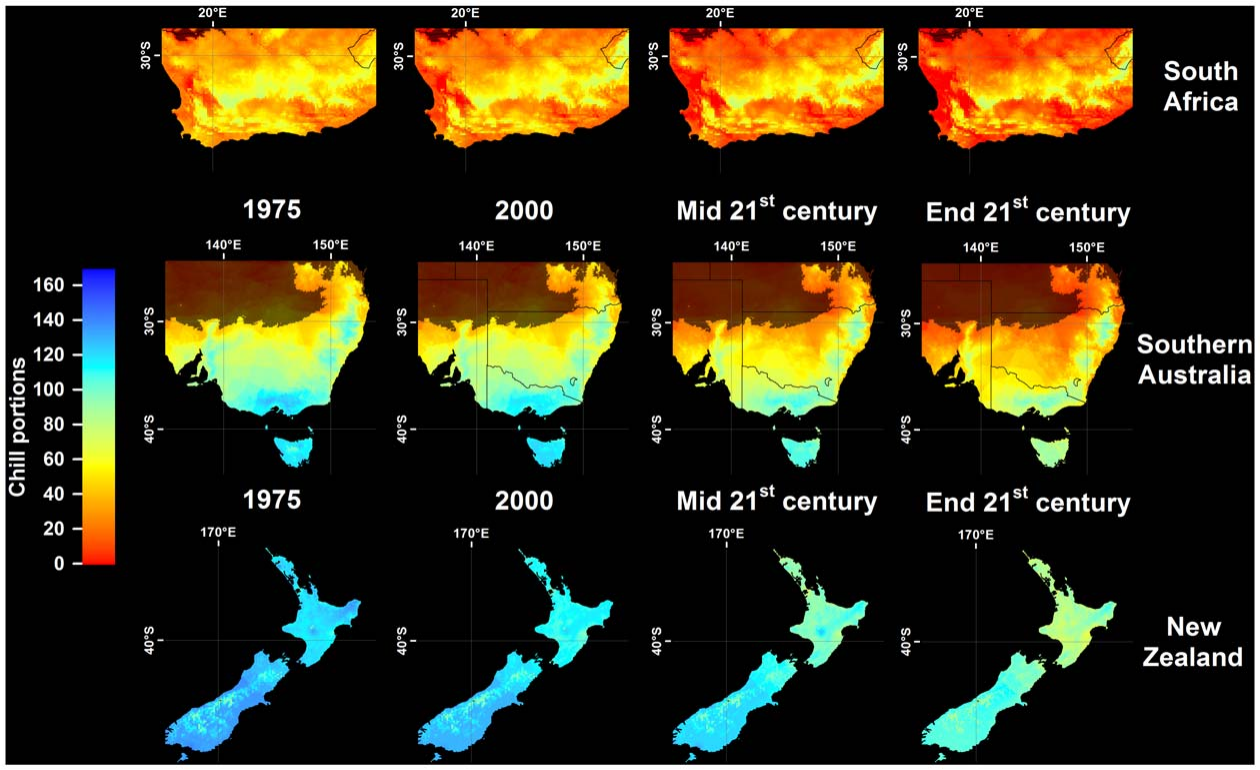
Modeled and projected Safe Winter Chill in South Africa, Southern Australia and New Zealand, for 1975, 2000, the middle of the 21^st^ century (middle), and the end of the 21^st^ century (bottom). For each point in time, results are averaged over three greenhouse gas emissions scenarios and three Global Climate Models. Areas that are more than 5° away from the closest weather station, and areas with mean annual temperatures >20 or <°0C are shaded.

Trends in site-specific projections for different growing regions varied substantially (site diagrams in Figs. 1–4). Most warm growing regions of temperate fruits and nuts are expected to experience decreasing winter chill, regardless of the emissions scenario or climate model used. The Sacramento Valley in California, the Southeastern United States, Chile's Valle Central, Yunnan Province in China, as well as South and Southwestern Australia are all projected to lose winter chill. This will likely require growers to transition to different species or cultivars than are grown today or to develop management practices that can help overcome shortages in winter chill. The highest losses relative to current winter chill levels occurred in Morocco, Tunisia, Israel, in the Cape region of South Africa and, for some GCMs, in the highlands of Kenya and Ethiopia. In these regions, climate change is likely to severely challenge current production systems, some of which already rely on cultural measures such as rest-breaking chemicals and artificial defoliation.

Cool regions are less likely to experience decreasing winter chill. Growing regions in Germany, the United Kingdom, the Midwestern United States (Fig. 7), Northern China and Central Asia are projected to see little change in SWC levels. Southern France (Fig. 6) and New Zealand (Fig. 8) may experience slight but likely insignificant losses. The coldest current growing regions (e.g. the Okanagan Valley in Canada, Southern Sweden and Eastern Europe) are expected to see more winter chill in the future. Whether these changes will require growers to adapt is currently unclear and is likely to depend more on the effects of summer warming than on winter temperatures.

**Figure 9:**
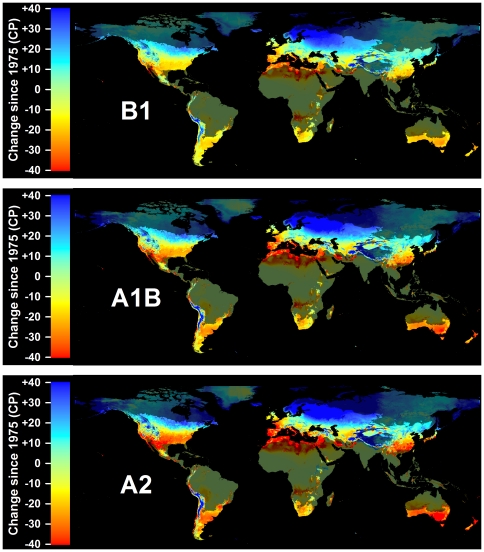
Projected losses in Safe Winter Chill at the end of the 21^st^ century compared to 1975, for three greenhouse gas emissions scenarios: B1 (top), A1B (middle) and A2 (bottom). For each scenario, results are averaged over projections from three Global Climate Models. Areas that are more than 5° away from the closest weather station are shaded, because interpolated results are unreliable.

In addition to the time period analyzed, the amplitude of expected changes also depended on the greenhouse gas emissions scenario. The A2 scenario consistently projected the greatest changes in winter chill, followed by the A1B scenario and the B1 scenario. If emissions are curbed to levels assumed in the B1 scenario, few growing regions are likely to see decreases by more than 20 CP by the end of the 21^st^ century (Fig. 9). If business-as-usual emissions continue (A2 scenario), many subtropical regions will see chilling declines up to 40 CP, which can be expected to disrupt production systems.

**Figure 10:**
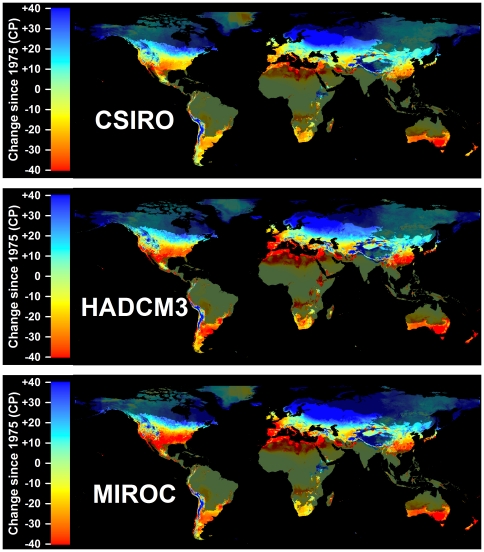
Projected losses in Safe Winter Chill at the end of the 21^st^ century compared to 1975, for three Global Climate Models: CSIRO (top), HADCM3 (middle) and MIROC (bottom). For each scenario, results are averaged over projections for three greenhouse gas emissions scenarios. Areas that are more than 5° away from the closest weather station are shaded, because interpolated results are unreliable.

The choice of the climate model also influenced model results. The MIROC model produced the greatest changes, followed by HADCM3 and CSIRO (Fig. 10). Within the same time period and emissions scenario, mean absolute differences between winter chill levels projected by CSIRO and HADCM3 were always smaller than for comparisons of either model with MIROC projections (Table 1). By the end of the 21^st^ century, mean absolute differences between all modeled scenarios and historic SWC levels for 1975 were between 12.8 and 29.0 CP, on average over all grid cells. These levels of change indicate that vegetation that relies on winter dormancy will experience very different temperature cues in the future than it does now.

**Table 1:** Mean absolute differences (in Chill Portions) between different climate scenarios [combination of time, greenhouse gas emissions scenario (GHG) and Global Climate Model (GCM)], over all relevant 0.1°×0.1° pixels of the global Safe Winter Chill projections.

Time			1975	2000	mid 21^st^ century									end 21^st^ century								
	GHG				B1			A1B			A2			B1			A1B			A2		
		GCM			C	H	M	C	H	M	C	H	M	C	H	M	C	H	M	C	H	M
**1975**				6.7	10.0	10.5	15.5	11.1	14.5	17.5	12.8	15.7	17.5	12.8	18.1	20.9	17.8	22.5	27.3	23.3	26.6	29.0
**2000**			6.7		7.5	7.2	11.9	6.2	9.5	13.9	7.7	10.6	13.7	9.2	14.0	17.1	13.8	18.1	23.5	18.6	22.6	24.8
**mid 21^st^ century**	**B1**	**C**	10.0	7.5		4.0	6.4	4.4	6.1	8.3	4.9	6.5	9.1	3.4	8.4	11.4	8.1	13.4	18.0	13.9	17.3	20.0
		**H**	10.5	7.2	4.0		6.0	4.9	5.2	7.9	5.3	6.5	8.9	3.8	7.8	11.0	8.1	12.8	17.5	13.6	16.8	19.5
		**M**	15.5	11.9	6.4	6.0		7.7	5.2	3.1	7.0	6.0	5.5	4.5	3.6	5.9	5.5	8.4	12.4	9.1	12.1	14.6
	**A1B**	**C**	11.1	6.2	4.4	4.9	7.7		4.7	9.3	2.5	5.4	9.7	4.7	9.2	12.1	8.8	12.9	18.3	13.2	17.3	19.5
		**H**	14.5	9.5	6.1	5.2	5.2	4.7		6.5	4.4	2.8	7.4	4.8	5.8	8.9	7.0	9.0	14.8	9.6	14.0	15.9
		**M**	17.5	13.9	8.3	7.9	3.1	9.3	6.5		7.8	6.5	5.5	5.9	4.0	3.7	4.6	6.7	10.2	7.3	10.0	12.7
	**A2**	**C**	12.8	7.7	4.9	5.3	7.0	2.5	4.4	7.8		4.4	8.9	4.3	7.8	10.4	7.0	11.2	16.5	11.3	15.5	17.7
		**H**	15.7	10.6	6.5	6.5	6.0	5.4	2.8	6.5	4.4		7.4	5.1	5.7	8.5	6.0	8.1	14.0	8.5	12.9	15.0
		**M**	17.5	13.7	9.1	8.9	5.5	9.7	7.4	5.5	8.9	7.4		7.4	6.0	7.2	7.0	8.8	12.4	9.4	11.9	14.2
**end 21^st^ century**	**B1**	**C**	12.8	9.2	3.4	3.8	4.5	4.7	4.8	5.9	4.3	5.1	7.4		5.8	8.8	5.4	11.0	15.4	11.3	14.6	17.5
		**H**	18.1	14.0	8.4	7.8	3.6	9.2	5.8	4.0	7.8	5.7	6.0	5.8		5.2	4.9	6.9	10.6	7.9	10.3	13.2
		**M**	20.9	17.1	11.4	11.0	5.9	12.1	8.9	3.7	10.4	8.5	7.2	8.8	5.2		5.3	5.9	6.8	5.2	8.0	9.5
	**A1B**	**C**	17.8	13.8	8.1	8.1	5.5	8.8	7.0	4.6	7.0	6.0	7.0	5.4	4.9	5.3		7.5	10.8	6.8	10.1	13.2
		**H**	22.5	18.1	13.4	12.8	8.4	12.9	9.0	6.7	11.2	8.1	8.8	11.0	6.9	5.9	7.5		7.7	4.9	6.8	8.0
		**M**	27.3	23.5	18.0	17.5	12.4	18.3	14.8	10.2	16.5	14.0	12.4	15.4	10.6	6.8	10.8	7.7		7.5	6.2	4.2
	**A2**	**C**	23.3	18.6	13.9	13.6	9.1	13.2	9.6	7.3	11.3	8.5	9.4	11.3	7.9	5.2	6.8	4.9	7.5		8.0	8.0
		**H**	26.6	22.6	17.3	16.8	12.1	17.3	14.0	10.0	15.5	12.9	11.9	14.6	10.3	8.0	10.1	6.8	6.2	8.0		7.1
		**M**	29.0	24.8	20.0	19.5	14.6	19.5	15.9	12.7	17.7	15.0	14.2	17.5	13.2	9.5	13.2	8.0	4.2	8.0	7.1	

GCMs: C – CSIRO; H – HADCM3; M – MIROC.

## Discussion

Our projections indicate that most warm growing regions will experience severe declines in Safe Winter Chill over the course of the 21^st^ century. In contrast to this, cool regions may not see much change, because reductions in winter chill due to warming are compensated for by chilling gains caused by less frequent frost. For cold growing regions, these opposing trends play out to result in more winter chill in response to warming.

Among these changes, reduced winter chill is likely to have the most severe consequences for fruit production. Lack of winter chill can delay or prevent flowering, lead to staggered bloom, and cause various forms of anomalous growth [26], [27]. Anecdotal evidence of this has been reported from various growing regions but seldom found its way into the literature.

Increases in winter chill in cold areas are less likely to lead to disruptions in fruit production, but even there, a mismatch between the chilling requirements of common species and cultivars and available winter chill could cause some problems for fruit and nut growers. In all areas, however, even in those where no changes in winter chill are projected, other manifestations of climate change are also likely to affect fruit and nut production. Plantations may be impacted by changes in rainfall, changes to summer and spring heat or increases in pest pressure due to faster reproduction of ectothermic pest organisms [28]. Due to all these additional effects of climate change, we do not attempt to predict future yields, but focus on pointing out potential problems due to lack of winter chill.

Even assuming no additional changes due to climate change, modeling the effect of changes in winter chill on crop yields is not possible at present. In spite of two centuries of research on winter chill, it is still unclear what happens during chilling accumulation, and how exactly this process is influenced by ambient temperatures. There may also be other environmental factors, such as relative humidity, photoperiod or the Red/Far Red light ratio [29], that impact the breaking of dormancy but are not recognized in any dormancy models for tree crops. All chilling models, including the Dynamic Model, were developed to assist fruit and nut growers in selecting appropriate species and cultivars, rather than describing a biological process with scientific accuracy. They are all empirically derived rather than based on a functional understanding of the dormancy process.

Lack of knowledge about the chilling requirements of most species and cultivars, in appropriate units, also precludes detailed projections of future yields or suitable ranges. Most growers and researchers have used Chilling Hours to quantify chilling requirements, but this model has been shown to perform poorly in warm regions [16], [17], [18], [19], [20] and to be very sensitive to climate change [15]. Estimates of chilling requirements, when given in Chilling Hours, must also be adjusted before they are useful in a different location or in a warmer climate [21]. Existing lists of species-specific chilling requirements are thus of limited value for estimating future ranges of cultivars and species. Estimates in Chill Portions are less widely available, and we are not aware of a comprehensive list that compiles them. Research efforts are needed to close the pertinent knowledge gaps and allow quantitative projections of the effect that changes in winter chill will have on fruit and nut production.

As much as precise quantitative projections are impossible, changes to available winter chill and summer heat will likely change the suitable ranges of many tree crops, and it seems likely that many growing regions will become unsuitable for the cultivars that are currently produced. However, whether or not tree crops will actually be moved to cooler climates will depend on many factors, such as availability of land and critical infrastructure, land tenure and competition with other crops. For example, the ecological niche of many fruits and nuts in the Western United States is likely to move north, from California's Central Valley towards Northern California, Oregon and Washington. These new potentially suitable areas have adverse topography, poorer soils, and limited water availability compared to the Central Valley, making the economic viability of production there questionable. Similarly, other regions, such as parts of Scandinavia, Canada and Siberia, that could potentially become more suitable for these tree crops, may be limited by cold winters, lack of summer heat, or adverse photoperiodic conditions. Production potential of tree crops under many of these novel conditions expected in the future has not been studied sufficiently to be discussed here.

To a certain extent, adaptation to changes in winter chill will be possible. Agrochemicals have been developed to artificially break a tree's dormancy during the later stages of chilling accumulation [30], [31], irrigation and shading may influence orchard microclimates favorably [32], and other cultural practices, such as artificial defoliation [33], also have potential for reducing chilling requirements. Inclusion of low chilling requirements as an explicit target in breeding programs is likely to produce cultivars that will remain suitable in a warmer future. It will be necessary, however, to intensify efforts to develop such adaptation strategies. In particular breeding programs for low-chilling cultivars, which can take decades to produce useable results, need more attention and more resources.

The economic cost of climate change incurred by fruit and nut growers could be substantial. Many businesses may be confronted with the decision to either abandon their production or adapt as well as possible to altered climatic conditions. Applying adaptation treatments could be economically unviable. And even if crops move towards more suitable climatic zones, most small orchard operations lack the capital to move their production to a different area, potentially impacting many livelihoods.

Natural plant communities respond to similar temperature cues as fruit and nut trees and will likely be affected as well by changes in the amount of available winter chill. It seems very likely that the projected decreases in winter chill in the Subtropics, but also the increases in the colder regions, will affect local natural vegetation, potentially impacting the suitable areas for many plants. Rapid climate-driven changes in plant communities at the local scale have already been reported [34]. Most studies that have investigated climate change effects on large numbers of plant species have found that most species showed advances in spring phenology, indicating that the impact of reduced chilling is compensated, in most cases, by increases in spring heat [35], [36], [37]. However, the same studies include a sizeable number of species, which show an opposite trend - delayed spring phenology in response to increases in temperature. For meadow and steppe vegetation on the Tibetan Plateau, Yu et al. [38] have recently shown a clear correlation of such a delay with increases in winter temperature, implicating lack of winter chill as the cause of the delay.

### Conclusion

Even under the most conservative emissions scenario examined here the projected substantial decreases in winter chill would negatively impact productivity of current cultivars and viability of fruit and nut industries in warm growing regions. Chemical, mechanical and physical methods to compensate for a loss of chilling are available but add significantly to production costs. A shift to new production areas may be possible but would incur substantial infrastructure costs. Moreover, production viability depends on many critical factors in addition to climatic suitability, which may not be available in areas with suitable future climates. The most viable approach to adapt to climate change in deciduous fruit and nut species is through the development of new cultivars that are more productive under lower chill conditions. This will require investment in efforts to understand the biological basis for chilling, to develop better models that relate environmental cues with yield and phenology, as well as renewed emphasis on breeding programs.

## Materials and Methods

### Weather data

Daily temperature and rainfall records were downloaded for all 11,361 available weather stations at the National Climatic Data Center [22]. This dataset was filtered, removing all stations that had less than 5000 daily records between 1973 and 2002, and excluding all stations with more than 25% of daily minimum or maximum temperatures or 50% of daily rainfall data missing. For the remaining 5078 weather stations, all available temperature and precipitation records were used to calculate site parameters for use in the LARS-WG stochastic weather generator [23], [24]. A stochastic weather generator (WG) is a model which, after calibration of site parameters with observed weather at that site, is capable of simulating synthetic time-series of daily weather that are statistically similar to observed weather [39]. By altering the site parameters of the WG using changes in climate predicted from a global climate model (GCM), it is possible to generate synthetic daily weather for the future. WGs are extensively used as a computationally inexpensive tool to produce daily site-specific climate scenarios for impact assessments of climate change [40], [41], [42], [43]. Because the generation of daily weather is dependent on the site-specific duration of wet and dry spells, the modeling procedure required precipitation records in addition to temperatures.

Following Luedeling et al. [8], for each station we evaluated the entire weather record for all days between 1973 and 2002, calculating separate linear regression equations between time (in years) and the minimum temperature, maximum temperature and precipitation for each month of the year. We then used these regression equations to develop climate scenarios representing typical climatic conditions in 1975 and 2000. These scenarios do not represent actually observed temperatures and precipitation in these years, but rather typical conditions at these times that are representative of long-term trends over the calibration period (1973–2002). The climate scenarios contain the mean deviation of monthly minimum and maximum temperatures and precipitation from the mean of the calibration period for each station.

Using the same method, we evaluated the weather record for 18 future scenarios, based on projections by three Global Climate Models (GCMs; MIROC3.2 (medres), UKMO-HadCM3 and CSIRO-Mk3.0) that had been statistically downscaled to a 0.5 degree resolution using the CRU TS 2.0 data set to calibrate the downscaling (R. Neilson, unpublished data). Using the Climate Wizard tool (www.climatewizard.org; ref. [25]), we extracted projected minimum and maximum temperatures and precipitation for three greenhouse gas emissions scenarios of the IPCC Special Report on Emissions Scenarios [44]: the A2 scenario (continually increasing rate of greenhouse gas emissions), the A1B scenario (emissions leveling off at mid 21^st^ century) and the B1 scenario (global curbing of emissions over the 21^st^ century). For each weather station, emissions scenario and GCM, mean monthly anomalies of minimum and maximum temperatures and precipitation relative to the calibration period were obtained for two periods of time: 2040–59 and 2080–2099, representing conditions at mid and end 21^st^ century. For several weather stations, mostly along coast lines, no GCM projection data were available. These stations were excluded, bringing the total number of weather stations down to 4293.

For each station and for each of the 2 past and 18 future scenarios, we then generated 101 years of synthetic daily weather data using a command line version of the LARS-WG weather generator [24]. Synthetic daily maximum and minimum temperatures were converted into hourly temperatures using the idealized temperature curve proposed by Linvill [9], [45]. Linvill's equations, which use a sine curve for daytime temperatures and a logarithmic decline curve for nighttime cooling, require sunset and sunrise hours, as well as daylength, as input parameters. These data were generated using equations by Spencer [46] and Almorox et al. [47]. Resulting from these processing steps were 101 years of synthetic hourly temperature for each weather station, representing typical weather conditions for each climate scenario.

### Winter chill

Based on the generated hourly temperature, we calculated winter chill for 100 winters for each weather station and climate scenario, with start and end dates of the winter season set to October 1^st^ and May 1^st^, respectively, for stations in the Northern Hemisphere, and to April 1^st^ and November 1^st^, respectively, for stations in the Southern Hemisphere. In a global analysis, effective times of winter chill accumulation often deviate from these dates, depending on local temperature curves. Since the Dynamic Model contains a self-regulating mechanism, which only allows accumulation of Chill Portions during times with appropriate temperatures, this variability should not affect the accuracy of our results.

For the resulting distribution over 100 winters, we then calculated Safe Winter Chill (SWC), the 10% quantile of the distribution [8]. This metric is more meaningful for growers than mean winter chill, because economic success of an orchard operation relies on fulfillment of chilling requirements in most years (e.g. 90% of all years), rather than in an average year. For all trees with lower chilling requirements than available SWC, the dormancy season should be sufficiently long and cold to allow fulfillment of tree-specific temperature needs. All data processing was implemented in JSL, the scripting language of JMP 8 (SAS Institute, Cary, NC, USA).

### Spatial analysis

**Figure 11:**
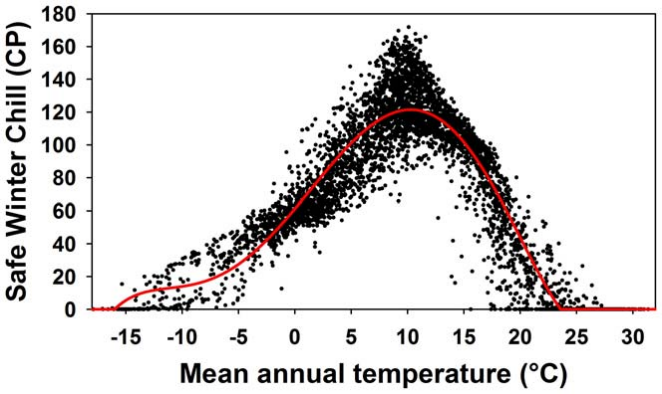
Correlation between mean annual temperature and modeled Safe Winter Chill for the year-2000 scenario. The red line indicates the equation used to correct for unaccounted for variation in temperature during spatial interpolation of site-specific Safe Winter Chill estimates.

Based on estimates of SWC for every weather station, SWC was spatially interpolated for each climate scenario, using 12-neighbor Kriging with a spherical semivariogram at 0.1 degree spatial resolution (ArcGIS 9.3, ESRI, Redlands, CA, USA). A simple interpolation based on only the available weather stations would not be a very accurate representation of winter chill around the world, because temperatures at many locations may differ substantially from those of the closest weather station, which is often far away. We therefore used a high-resolution temperature dataset obtained from the WorldClim database [48] to correct for temperature variation that was not accounted for by the simple interpolation procedure. We calculated mean annual temperatures at a spatial resolution of 1/24 degree from monthly mean temperatures for the year 2000 given in the database. Because mean annual temperature explains much of the variation in winter chill [21], this dataset was useful for correcting the chilling estimates. To do this, we used the same Kriging procedure as for the winter chill estimates to interpolate a temperature surface from mean annual temperatures at all weather stations. The resulting grid represents the temperatures that correspond to the interpolated chill portion surface (SWC_int_). Subtracting this grid from the original dataset of mean annual temperatures produced an estimate of the temperature variation that was not accounted for in the original interpolation of chill portion values (T_diff_). For the correction, the effect of temperature on chill portion numbers was then estimated by a 5^th^ order polynomial regression between mean annual temperatures at all weather stations and the amount of safe winter chill calculated for them (Fig. 11). The resulting regression equation was:
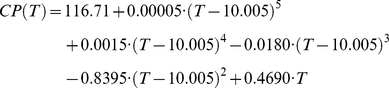
for −15.996<T<23.594, and CP(T) = 0 for temperatures outside this range. In this equation, T is the temperature and CP(T) is the corresponding number of chill portions. The final correction equation was then:
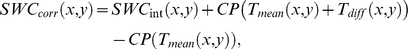
with T_mean_ being a Kriging surface calculated from mean station temperatures of the respective climate scenario, and x and y the longitude and latitude of each grid cell. SWC_corr_ is the temperature corrected estimate of safe winter chill. On all maps, areas where mean annual temperatures were below 0°C or above 20°C were shaded, because such regions are not suitable for the production of fruits and nuts with chilling requirements. Likewise, all areas that were further than 5° away from the closest useable weather station were shaded, because interpolation results for such grid cells were deemed unreliable. All gridded SWC layers are available from http://treephenology.ucdavis.edu/.

### Scenario evaluation

From the surfaces of safe winter chill, site-specific results were extracted for 24 point locations representing important growing regions around the world. Comparing safe winter chill estimates for different combinations of GCM, GHG emissions scenario and point in time provides an impression of the agreement between scenarios, on a case-study basis. We also evaluated differences between scenarios based on the entire distribution over all relevant grid cells (excluding all that were shaded in the maps). Because differences between models in site specific estimates can be both negative and positive, we evaluated the mean absolute difference among all grid cells. The results are indicative of the agreement between models.
